# Still dealing with paracetamol overdoses: epidemiology and quality of data collected in the Scottish health system from 2010 to 2023

**DOI:** 10.1093/pubmed/fdaf076

**Published:** 2025-07-04

**Authors:** Nasloon Ali, Andrew Radley, Giorgia De Paoli

**Affiliations:** School of Health Sciences, University of Dundee, 11 Airlie Pl, Dundee DD1 4HJ, UK; School of Health Sciences, University of Dundee, 11 Airlie Pl, Dundee DD1 4HJ, UK; School of Health Sciences, University of Dundee, 11 Airlie Pl, Dundee DD1 4HJ, UK

**Keywords:** public health, research

## Abstract

Despite the legislation implemented in the UK in 1998 on paracetamol pack restriction, overdoses remain high. In this work, data on Accident and Emergency (A&E) (2020–2023) attendances for paracetamol overdoses collected via Freedom of Information requests have been investigated. Additionally, hospital admissions (2010–2021) for paracetamol overdoses obtained from Public Health Scotland have been preliminary analyzed to understand the phenomenon within the Scottish community. Data on A&E attendances provided were limited and showed discrepancies amongst the different Scottish Health Boards for type, quality and amount of data recorded, thus preventing accurate figures. Overall, average number of absolute hospital admission was ⁓5800 per year, with the highest numbers in 2013, 2019, and 2021. When admissions were population-adjusted (with population data from National Records of Scotland), they revealed a different trend. From 2017 onward, smaller Health Boards exhibited higher rates than the larger ones. There is an urgent need for streamlined publicly accessible data and harmonized data collection across Health Boards. This approach would ultimately lead to the development of tailored, new interventions (or the adaptation of existing ones) to promote safe use of paracetamol.

## Introduction

UK paracetamol pack size restriction legislation was introduced in 1998,^1^ reducing deaths and liver transplants by 61% (1998–2009),^2–6^ yet hospital admissions in Scotland from paracetamol poisoning (intentional or accidental) remain high (c.5800 each year).^7^

This issue is gaining political attention, as shown by a Scottish Parliament member's inquiry to Public Health Scotland (PHS) about high admissions and deaths from intentional paracetamol overdoses.^7^

This study aimed to use Scottish data on Accident & Emergency (A&E) attendances (2020–2023) and hospital admissions (2010–2021) for paracetamol-only overdosing (intentional or accidental) to underpin the extent of such overdose nationwide.

## Methods

### Data sources

Public domain data or freely available upon request.

### A&E attendances for paracetamol overdoses

A&E attendances (2020–2023) categorized by age groups (15-year-old or younger and 16-year-old or older), intent (intentional or accidental), and contributing factors (e.g. dental pain, back pain) were obtained from each Scottish Health Board (HB) via Freedom of Information (FOI) request between December 2023 and January 2024 (Responses in Supplementary File 1).

### Hospital admissions for paracetamol overdoses

Data were from Scotland Accident and Emergency^8^ and the General Acute Inpatient and Day Case—Scottish Morbidity Record (SMR01)^9^ and were extracted using ‘The International Classification of Diseases’ (ICD) code, T39.1 code (poisoning by 4-aminophenol derivatives). Population data were from the National Records of Scotland (NRS, Population Estimates)^10^ database. Period considered: from 2010 to 2021.

### Statistical analyses/data visualization

Data were arranged in Microsoft Excel (as per original format provided). Rates and prevalences were calculated using cases/population numbers for HB, age categories and intentional versus accidental overdoses. Standardization and normalization of data was not possible due to missing data in certain HBs. Admissions data calculations (absolute total and average for paracetamol overdoses -intentional/accidental—per HB/year, population extraction per HB/year, admission rates per population, mean, median, standard deviation, range) were done in Stata SE 18. Data visualization was achieved using RAWGraph,^11^ and Adobe Illustrator.^12^

## Results

### A&E attendances for paracetamol overdoses

**Figure 1:**
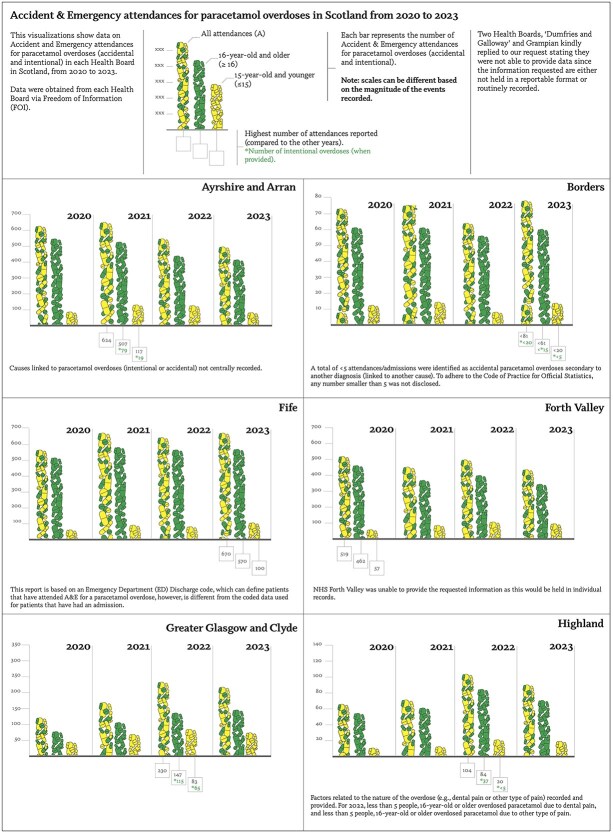

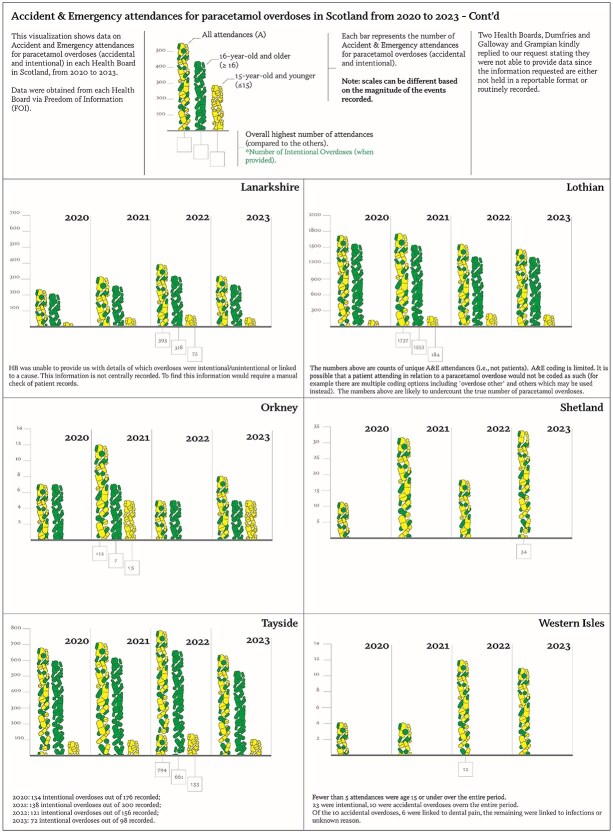
Figure with bar charts showing accident and emergencies (a&E) attendances for paracetamol overdoses (accidental and intentional) across twelve Scottish health boards from 2020 to 2023. Two health boards (Dumfries and Galloway and Grampian) did not provide data. Bars are categorized by year, showing reporting numbers for all attendances, 16 and older individuals’ attendances, and 15 and younger individuals’ attendances. Highest attendance values noted per year, reporting the number below the corresponding bar. When available, intentional paracetamol overdoses are reported, marked by an asterisk. Attendance counts ranged from fewer than 5 to over 1700. Counts ˂5 are suppressed for data protection.

Data received showed some discordance around the type, quality and amount of data recorded, therefore we opted for a narrative approach contextualizing the findings (see Fig. 1) and reporting them holistically.^13^

Increasing numbers of overdoses were seen in 2020, 2021, and 2022, followed by a decrease in 2023. The counts reflect unique attendances, though some patients may have had multiple visits. Cases may also have been coded under broader categories like ‘overdose other’, likely underestimating true paracetamol overdose events.

#### Intentional/accidental paracetamol overdoses

Some HBs recorded data around the overdose intent (intentional and accidental -Ayrshire and Arran, Greater Glasgow and Clyde, Highland-), for other HBs, this information was held centrally thus not readily available, or withheld to maintain confidentiality.^14^

#### 16-year-old and older

For this group, Ayrshire and Arran highest percentage of intentional paracetamol overdoses were 18.65% in 2020 (520 cases), and lowest 9.57% in 2023 (397 cases). Greater Glasgow and Clyde highest percentage was 81.12% in 2023 (214 cases) and lowest 65.79% in 2020 (76 cases). Highland recorded the highest percentage of 44.05% in 2022 (84 cases) and lowest of 27.14% in 2023 (70 cases).

#### 15-year-old and younger

When recorded, deliberate overdoses ranged from 5% to 16% (5–19 cases) in Ayrshire and Arran (2020 and 2021 the highest), and 42%–54% (8–6 cases) in the Highland.

### Factors linked to paracetamol overdoses

Dental pain or infections were the most prominent factors amongst the two HBs providing this information. In the Western Islands, dental pain represented 60% of 10 accidental paracetamol overdoses (2020–2023).

### Hospital admissions for paracetamol overdoses

#### Overall admissions

**Figure 2:**
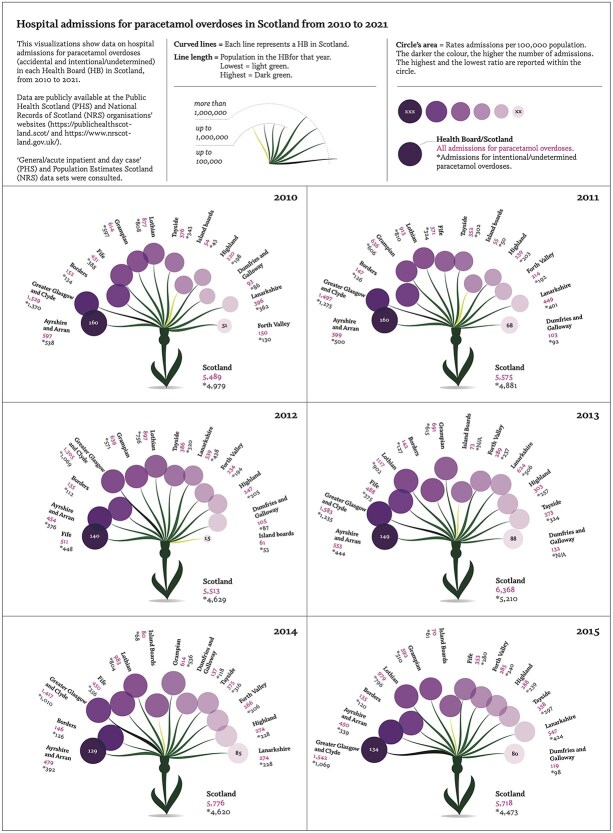

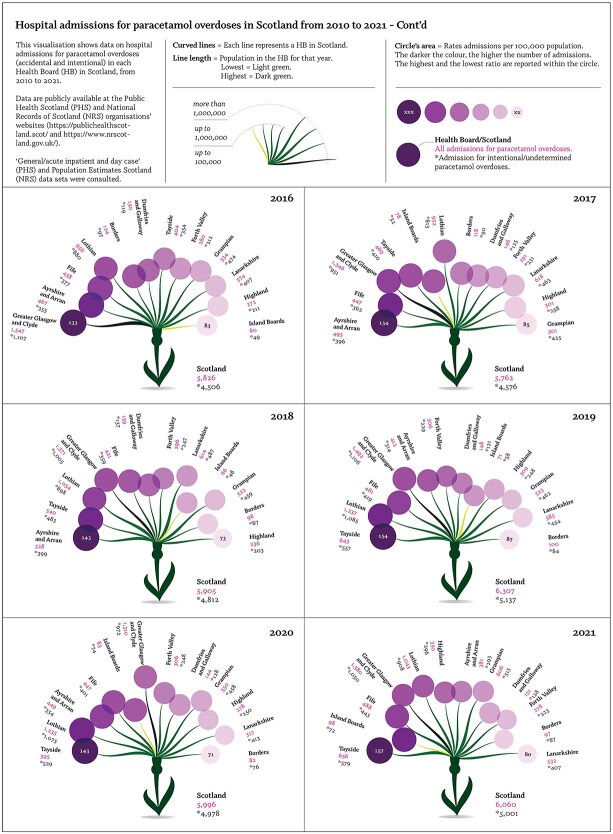
Figure with stylized Scottish thistle illustrating paracetamol overdose hospital admissions from 2010 to 2021, by Scottish health board. Curved line length represents population count, circle size represents the rate of overdoses per 100 000 individuals. The darker the colour of the circle, the higher the number of admissions. Below each health board is reported the overall number of paracetamol overdose admissions, and the number of intentional overdoses (marked with an asterisk). This information is also reported for all of Scotland for each year.

Data were reported via creative data visualization approach (Fig. 2). The thistle, a national symbol of Scotland, was used to represent paracetamol overdoses’ hospital admissions. We reported total number of admissions and intentional overdoses for each HB and for Scotland, as well as the highest and lowest rate of hospital admissions per 100 000 people. A simplified version can be found in Supplementary File 2.

Data for Orkney, Shetland and Western Isles were grouped under ‘Island Boards’ by PHS to preserve data confidentiality.

Overall, average number of hospital admission for paracetamol overdoses from 2010 to 2021 was ⁓5800 admissions a year. 2013, 2019, and 2021 shown the highest numbers of admissions (6368 and 6307 and 6060, respectively); 2010, 2012, and 2011 the lowest admissions (5489, 5513, and 5575).

As expected, HBs with larger populations had highest counts of paracetamol overdoses. However, when admissions were population-adjusted, they revealed a different trend. From 2017 onward, smaller HBs exhibited higher rates than the larger ones.

#### Intentional paracetamol overdoses

Data provided by PHS were categorized as either ‘accidental’ or ‘intentional or undetermined’. Although we are unable to determine the specific number of intentional cases, the intentional/undetermined group numbers are concerning, ranging from 4506 cases (77.3%) in 2016 to 4979 cases (90.7%) in 2010.

#### Accidental paracetamol overdoses

The proportion of accidental overdoses ranged from 9.3% (in 2010) to 22.7 (in 2016). From 2010–2013, we observed a steady increase in the percentage, a trend worth considering for future research.

#### Paracetamol overdoses by age

Data from PHS were provided considering three age categories: under 15-year-old, 15–64-year-old and over 65-year-old.

Whilst these broad age groups pose a limit for insightful conclusions, a preliminary data distribution analysis showed that most overdoses events (87.53%) occurred within the 15–64-year-old group. Amongst these, accidental events accounted for 73.33%, and intentional/undetermined events for 90.65%. Findings not included in Fig. 1, as further analysis is necessary for meaningful insight.

## Discussion

### Main findings of this study

Administrative data provides a foundation for investigating paracetamol overdoses in Scotland (c.5800 per year) and developing/adapting targeted interventions to reduce the issue.

### What is already known on this topic

National interventions so far applied to reduce paracetamol overdoses have only had short-term positive impact on deaths and liver transplants.

### What this study adds

This study highlights inconsistencies in data collection across Scottish HBs, limiting data investigations.

### Limitations of this study

Unintentional overdoses lack a clear pattern and risk factor associations (such as information on the pandemic) could not be determined due to data unavailability.

## Conclusions

There is an urgent need for streamlined publicly accessible data and consistent data collection across HBs. This would help address the paracetamol epidemic, allowing for tailored, more effective interventions.

## Supplementary Material

Supplementary_file_1_Paracetamol_overdoses_FOI_responses_fdaf076

Supplementary_file_2_Hospital_admissions_for_paracetamol_overdoses_by_HB_fdaf076

Supplementary_text_fdaf076

## Notes

**Nasloon Ali**, Lecturer, Epidemiologist

**Andrew Radley**, Professor of Pharmaceutical Public Health, Pharmacist

**Giorgia De Paoli**, Lecturer, Pharmacist, Forensic Toxicologist
