# Supplementary material for: Still dealing with paracetamol overdoses: epidemiology and quality of data collected in the Scottish health system from 2010 to 2023
Source: J Public Health (Oxf). 2025 Jul 4;47(4):721–7. doi: 10.1093/pubmed/fdaf076 (PMC12669989; doi:10.1093/pubmed/fdaf076)
Supplement: Supplementary_file_1_Paracetamol_overdoses_FOI_responses_fdaf076 [file supplementary_file_1_paracetamol_overdoses_foi_responses_fdaf076.pdf]

Supplementary file 1:

Responses received from Scottish Health boards in relation to the Query submitted through the Freedom of Information act.

Queries submitted

1. Number of A&E admissions for paracetamol overdoses from 2020 to 2023 in the specific NHS health board
2. Age of people presenting at A&E with a primary diagnosis of paracetamol overdosing (above and below 15-year-old)
3. Is it possible to discriminate which ones were intentional and which were unintentional?
4. Is it possible to know if these overdoses were linked to a cause (e.g., dental pain, backpain…)

Table reporting information request/information received dates

NHS Health board	Request sent	Information received
NHS Ayrshire and Arran	19/12/2023	28/12/2023
NHS Borders	19/12/2023	23/01/2024
NHS Dumfries and Galloway	19/12/2023	05/01/2024
NHS Fife	19/12/2023	18/01/2024
NHS Forth Valley	19/12/2023	12/01/2024
NHS Grampian	19/12/2023	18/01/2024
NHS Greater Glasgow and Clyde	19/12/2023	17/01/2024
NHS Highland	19/12/2023	04/01/2024
NHS Lanarkshire	19/12/2023	12/01/2024
NHS Lothian	19/12/2023	11/01/2024
NHS Orkney	19/12/2023	04/01/2024
NHS Shetland	19/12/2023	04/01/2024
NHS Tayside	05/12/2023	18/12/2023
NHS Western Isles	19/12/2023	04/01/2024

Supplementary file 1:

Responses received from Scottish Health boards in relation to the Query submitted through the Freedom of Information act.

NHS Ayrshire and Arran

1.

All patients meeting above criteria Year	Number of Attendances
2020	594
2021	624
2022	517
2023	472

2.

All patients meeting above criteria categorised by age band Year	Age 15 and below	Age 16 and above
2020	74	520
2021	117	507
2022	99	418
2023	75	397

3.

All patients meeting above criteria and where the First diagnosis is recorded as self-poisoning or suicide risk. Year	Age 15 and below	Age 16 and above
2020	11	97
2021	19	79
2022	5	48
2023	9	38

4.

This information is not centrally recorded. It is not possible without reviewing each individual patient's case notes to provide the detail requested. We would need to identify these patients, retrieve and read through each health record to extract the relevant data. This would be burdensome on our limited resources and a time consuming exercise. We estimate this would take several hours and be out with the cost of compliance as this would exceed the £600 prescribed limit set in regulation 5 of the Freedom of Information (Fees for Required Disclosure)(Scotland) Regulations 2004. Therefore NHS Ayrshire & Arran is not obliged under section 12(1) of the Freedom of Information (Scotland) Act 2002 to comply with your request. You may wish to narrow the parameters of your request, which may bring the cost of compliance below the threshold. Any further request for information will be treated as a new Freedom of Information request. If you wish to narrow your request please contact me and we can discuss what could be provided within the cost limit.

SUPPLEMENTARY FILE 1:

RESPONSES RECEIVED FROM SCOTTISH HEALTH BOARDS IN RELATION TO THE QUERY SUBMITTED THROUGH THE FREEDOM OF INFORMATION ACT.

NHS Borders

---

1. The number of Emergency Department attendances for paracetamol overdoses is:

	Under 15yrs			Over 15yrs Over		
	Deliberate Self-Harm	Not Known Unintentional	Unintentional	Deliberate Self-Harm	Not Known Unintentional	Unintentional
2020	<5	8	0	20	37	<5
2021	<5	10	0	15	38	8
2022	<5	<5	0	12	40	<5
2023	<5	10	<5	15	41	<5

The number of admissions to the Borders General Hospital for paracetamol overdoses is:

	Under 15yrs		Over 15yrs Over	
	Deliberate Self-Harm	Unintentional	Deliberate Self-Harm	Unintentional
2020	8	<5	71	6
2021	9	<5	87	9
2022	<5	<5	39	<5
2023	13	<5	47	5

There was a total of <5 attendances/admissions identified as an accidental paracetamol overdose secondary to another diagnosis (linked to another cause). to provide further details of these could allow individuals to be identified and we would be in breach of the Data Protection Act 2018. We are therefore withholding all other data under Section 38(2)(ii) of the Freedom of Information (Scotland) Act 2002.

As the number of events in some areas are very small and in accordance with the Code of Practice for Official Statistics any number that is less than five, actual numbers and potentially identifiable information is withheld to help maintain patient confidentiality due to potential risk of disclosure. Further information is available in the ISD Statistical Disclosure Control Protocol.

Please note: There are differences in the numbers of ED attendances and BGH admissions and this is due to where the paracetamol overdose diagnosis is recorded.

NHS Dumfries and Galloway

Supplementary file 1:

Responses received from Scottish Health boards in relation to the Query submitted through the Freedom of Information Act.

NHS Dumfries and Galloway do not hold this information in a reportable format; therefore, this information is refused under section 17 of FOISA. There isn't a specific code for Paracetamol overdose, only general codes that could include other medications.

Under section 20 (1) of the Act, if you are dissatisfied with the way NHS Dumfries and Galloway has dealt with your request, you have a right to request a review of our actions and decisions in relation to your request, and you have a right to appeal to the Scottish Information Commissioner.

A request for review must be made within forty working days from 05 January 2024 and should, in the first instance, be in writing [redacted]. You must provide your name, an address for correspondence, details of your original request and why you want a review.

If our decision is unchanged following review and you remain dissatisfied with this, you have the right to make a formal appeal to the Scottish Information Commissioner. Requests for appeal should be made in writing to the [redacted]

NHS Fife

1.

Please note: This report is based on an Emergency Department (ED) Discharge code, which can define patients that have attended A&E for a paracetamol overdose, however, is different from the coded data used for patients that have had an admission. NHS Fife therefore is unable to answer any further questions using the ED Discharge code.

Year	Patient Age	ED Attendances
2020	Aged 15 Years and over	515
	Under 15 Years Old	53
2021	Aged 15 Years and over	579
	Under 15 Years Old	90
2022	Aged 15 Years and over	567
	Under 15 Years Old	75
2023	Aged 15 Years and over	570

Supplementary file 1:

Responses received from Scottish Health boards in relation to the Query submitted through the Freedom of Information act.

Year	Admissions	Total Attendances	Attendances>15	Attendances<=15
2020	333	519	462	57
2021	244	449	367	82
2022	280	493	394	99
2023	246	432	341	91
Total	1103	1893	1564	329

	Under 15 Years Old	100

NHS Forth Valley

Only data received. Not possible to obtain data around causes.

NHS Grampian

this information is not routinely recorded and that to try and obtain this information would require manual examination of every patient record, which is impractical and would exceed the cost limit under the Act; s.12(1) of the Act refers – Excessive cost of compliance.

NHS Greater Glasgow and Clyde

ED Presentations & Admissions for Paracetamol Overdoses

Ages 0-15						
	Accidental Poisoning		Intentional Poisoning		Unknown	
Attend Year	ED Attends	ED Admissions	ED Attends	ED Admissions	ED Attends	ED Admissions
2020	11	5	28	19		
2021	20	12	44	26	<5	0
2022	18	<5	65	39		
2023	14	5	57	41		

Ages 16+				
	Accidental Poisoning		Intentional Poisoning	
Attend Year	ED Attends	ED Admissions	ED Attends	ED Admissions
2020	26	<5	50	18
2021	32	5	71	26
2022	32	<5	115	35
2023	27	<5	116	42

SUPPLEMENTARY FILE 1:

RESPONSES RECEIVED FROM SCOTTISH HEALTH BOARDS IN RELATION TO THE QUERY SUBMITTED THROUGH THE FREEDOM OF INFORMATION ACT.

NHS Highland

**Paracetamol Overdose**	**2020**	**2021**	**2022**	**2023**
Total Patients Attending A&E	65	70	104	89

**Overdose**	**2020**	**2021**	**2022**	**2023**
Age 15 & under	11	10	20	19
Age 16 & above	54	60	84	70
Intentional Overdose	2020	2021	2022	2023
Age 15 & under	6	<5	<5	8
Age 16 & above	21	19	37	19
Overdose due to Dental pain	2020	2021	2022	2023
Age 15 & under	0	<5	0	0
Age 16 & above	9	<5	<5	5
Overdose due to other pain	2020	2021	2022	2023
Age 15 & under	0	0	0	0
Age 16 & above	<5	0	<5	<5

NHS Lanarkshire

**Attendances**	**Total**
2020	**236**

2021	**314**
2022	**393**
2023	**318**
**Grand total**	**1261**

Attendances by age	15 and under	16 and over	Total
2020	27	209	**236**
2021	58	256	**314**
2022	75	318	**393**
2023	55	263	**318**
**Grand total**	**215**	**1046**	**1261**

I regret to advise that I am unable to provide you with details of which overdoses were intentional/unintentional or were linked to a cause. This information is not centrally recorded. To find this information would require a manual check of patient records.

NHS Lothian

Answer:

Year	Aged younger than 15	Aged 15+	Total
2020	129	1593	1722
2021	184	1553	1737
2022	269	1336	1605
2023	206	1206	1412

Notes

The numbers above are counts of unique A&E attendances (i.e. not patients). Therefore, any given patient may be counted multiple times.
A&E coding is limited and it is possible that a patient may attend in relation to a paracetamol overdose and it would not be coded as such (for example there are multiple coding options including 'overdose other' and others which may be used instead). Therefore the numbers above are likely to undercount the true number of paracetamol overdoses

Note that it's not possible to provide information for the following aspects -

- *Is it possible to discriminate which ones were intentional and which were unintentional?*
- *Is it possible to know if these overdoses were linked to a cause (e.g., dental pain, backpain,…)*

This level of detail is not held in a centrally extractable format. Under the Freedom of Information Act NHS Lothian is not required to create new records to enable it to respond to your enquiry. This information is not collated or held in aggregate form and it would be necessary to review all case files relating to patients over the period you have requested to assemble the information you seek. Even if NHS Lothian did this – and there would be significant cost implications in doing so – it would be unable to respond in full to your request. The information requested is therefore exempt under section 12.1 – Cost.

NHS Orkney

The small size of the Orkney population and the small sample size result in some of the numbers involved being less than five means that the data is potentially patient-identifiable. It is therefore exempt under Section 38(1)(b) of the Freedom of Information (Scotland) Act 2002 and <5 has been noted in the relevant section.

Year Age under

15

Age 15 and

above

Intentional Unintentional

2020 0 7 <5 <5

2021 <5 7 <5 7

2022 0 5 <5 <5

2023 <5 <5 <5 <5

Year	Age under 15	Age 15 and above	Intentional	Unintentional
2020	0	7	<5	<5
2021	<5	7	<5	7
2022	0	5	<5	<5
2023	<5	<5	<5	<5

2. The requested information is not collated by NHS Orkney, therefore I must advise that in terms of

Section 17 of the Freedom of Information (Scotland) Act 2002, the information sought is not held.

NHS Shetlands

Year	No of Attendances
2020	11
2021	31
2022	18
2023	34
Grand Total	94

Responses are based on the number of A&E attendances where the presenting complaint or diagnosis includes Paracetamol Overdose.

Age of Patient	No of Attendances
15 and below	12
Above 16	82

30 of the 94 attendances were identified as intentional self harm.

4. Is it possible to know if these overdoses were linked to a cause (e.g., dental pain, back pain)

No

NHS Tayside

Supplementary file 1:

responses received from Scottish Health boards in relation to the Query submitted through the Freedom of Information act.

Year	Attendances at ED for Paracetamol Overdose
2020	667
2021	712
2022	794
2023 (to 12 December 2023)	632

NHS Western Isles

A1 2020: 5
2021: 5
2022: 12
2023 to 17/12/23: 11
A2 Fewer than 5 admissions were aged 15 or under over the entire period
A3 23 were intentional, 10 were accidental overdoses
A4 Of the 10 accidental overdose admissions, 6 were linked to dental pain, the remainder were linked to infections or reason unknown
Exemption Applied <5
We are unable to provide you with the detailed information requested above as we are bound by the laws of confidentiality in respect of individuals.
