# Supplementary material for: Still dealing with paracetamol overdoses: epidemiology and quality of data collected in the Scottish health system from 2010 to 2023
Source: J Public Health (Oxf). 2025 Jul 4;47(4):721–7. doi: 10.1093/pubmed/fdaf076 (PMC12669989; doi:10.1093/pubmed/fdaf076)
Supplement: Supplementary_file_2_Hospital_admissions_for_paracetamol_overdoses_by_HB_fdaf076 [file supplementary_file_2_hospital_admissions_for_paracetamol_overdoses_by_hb_fdaf076.pdf]

Supplementary file 2: Hospital admissions for paracetamol overdoses in Scotland from 2010 to 2021 by Health Board

This supplementary file show data on hospital admissions for paracetamol overdoses (accidental and intentional) in each Health Board (HB) in Scotland from 2010 to 2021. Values are expressed in rates of admissions per 100,000 population in the specific HB. Mean, Median, Standard Deviation, Range are also reported for each HB.

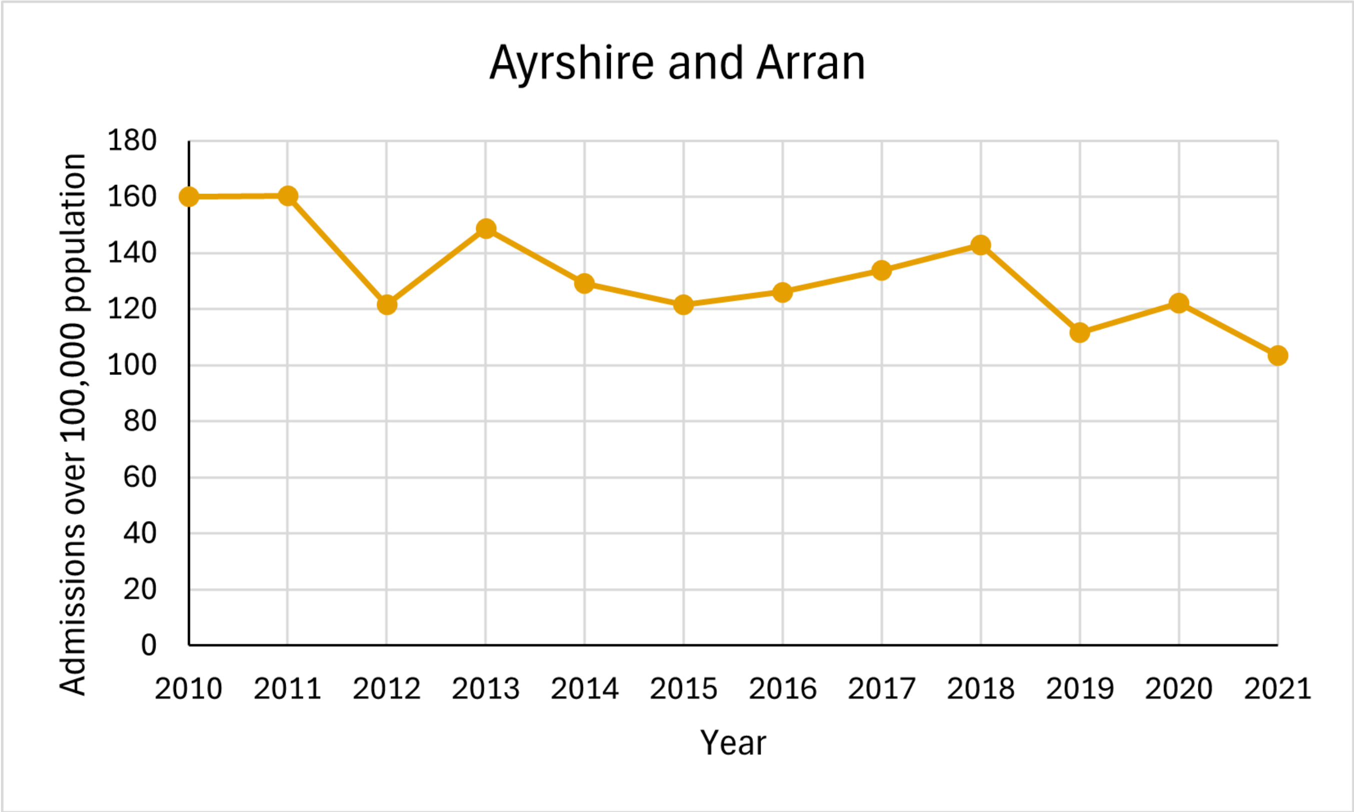

Mean: 132
Median: 128
Standard Deviation: 18
Range: 57

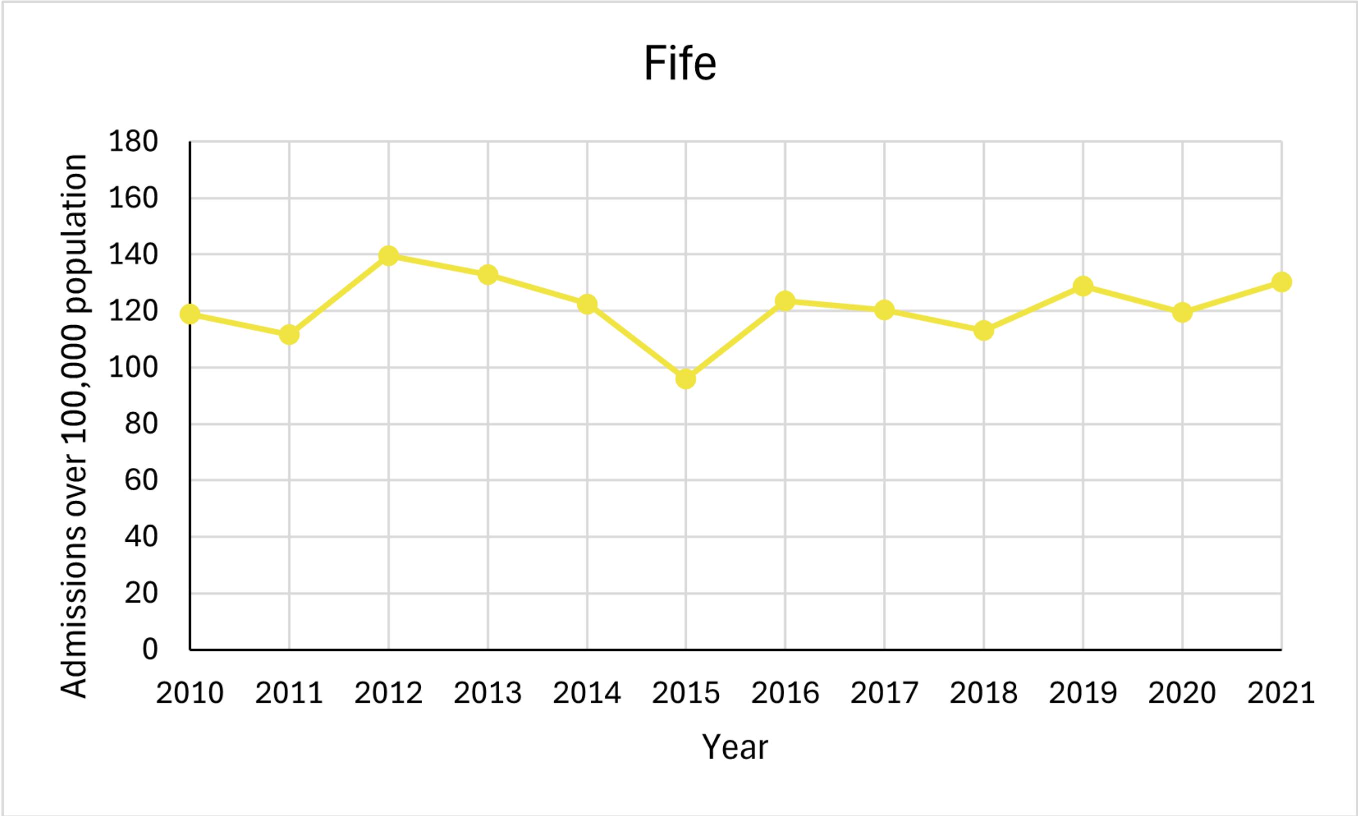

Mean: 121
Median: 121
Standard Deviation: 11
Range: 44

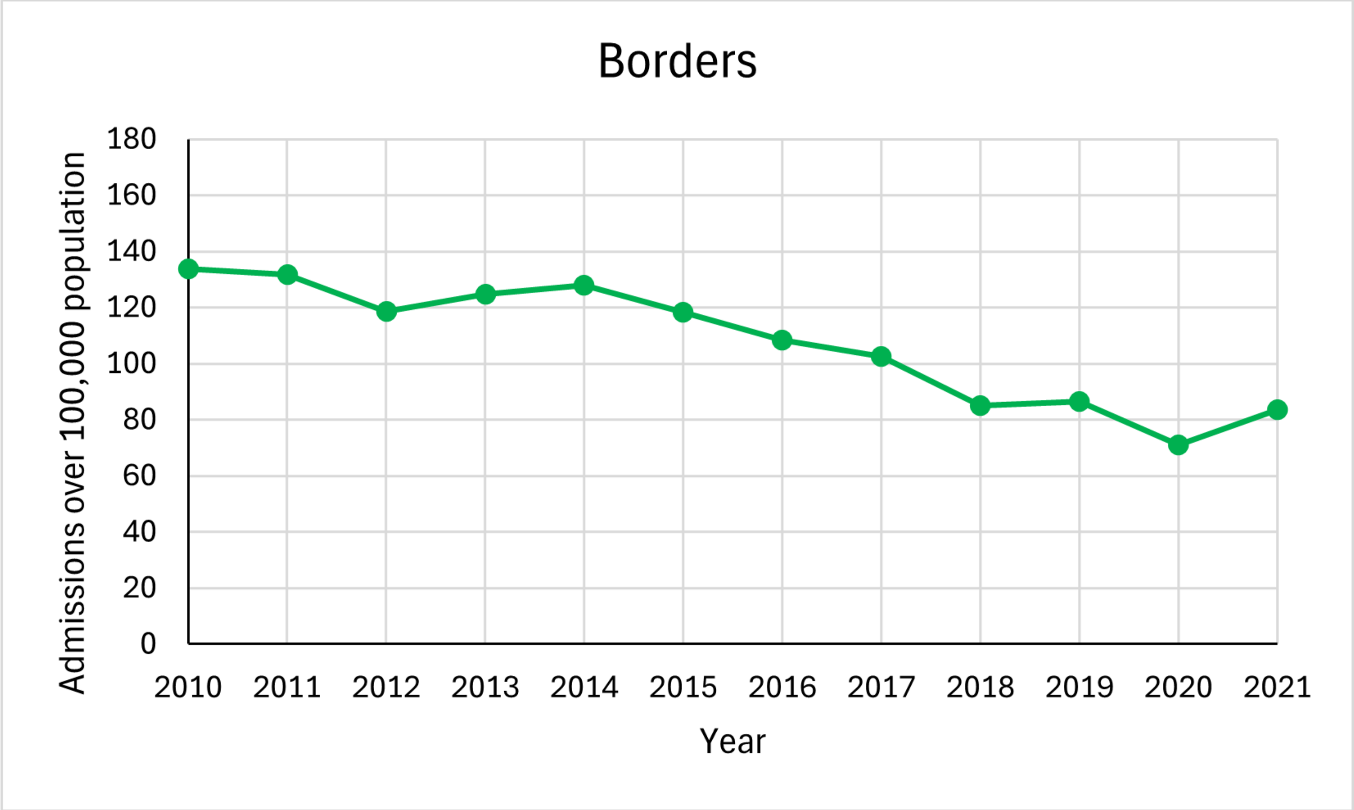

Mean: 108
Median: 113
Standard Deviation: 22
Range: 63

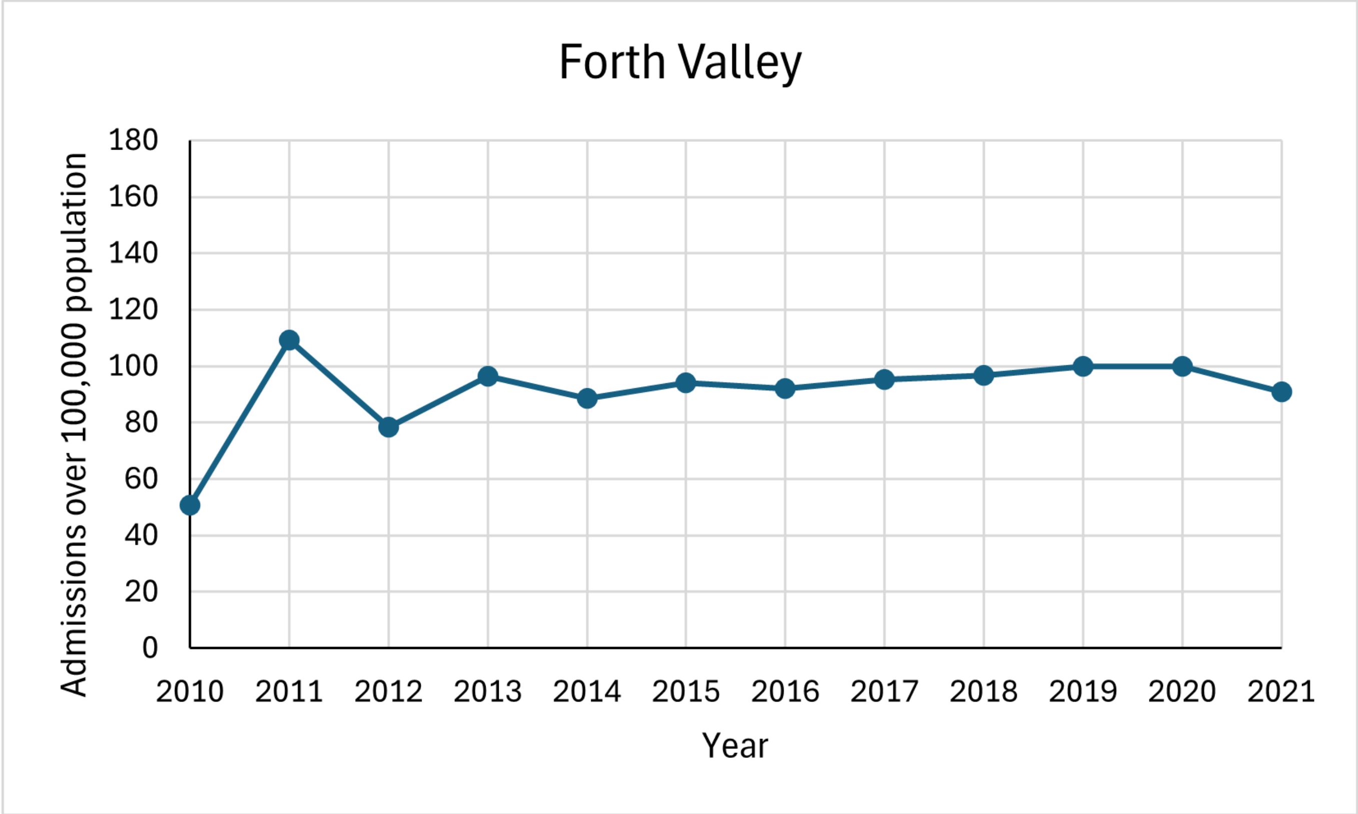

Mean: 91
Median: 95
Standard Deviation: 15
Range: 58

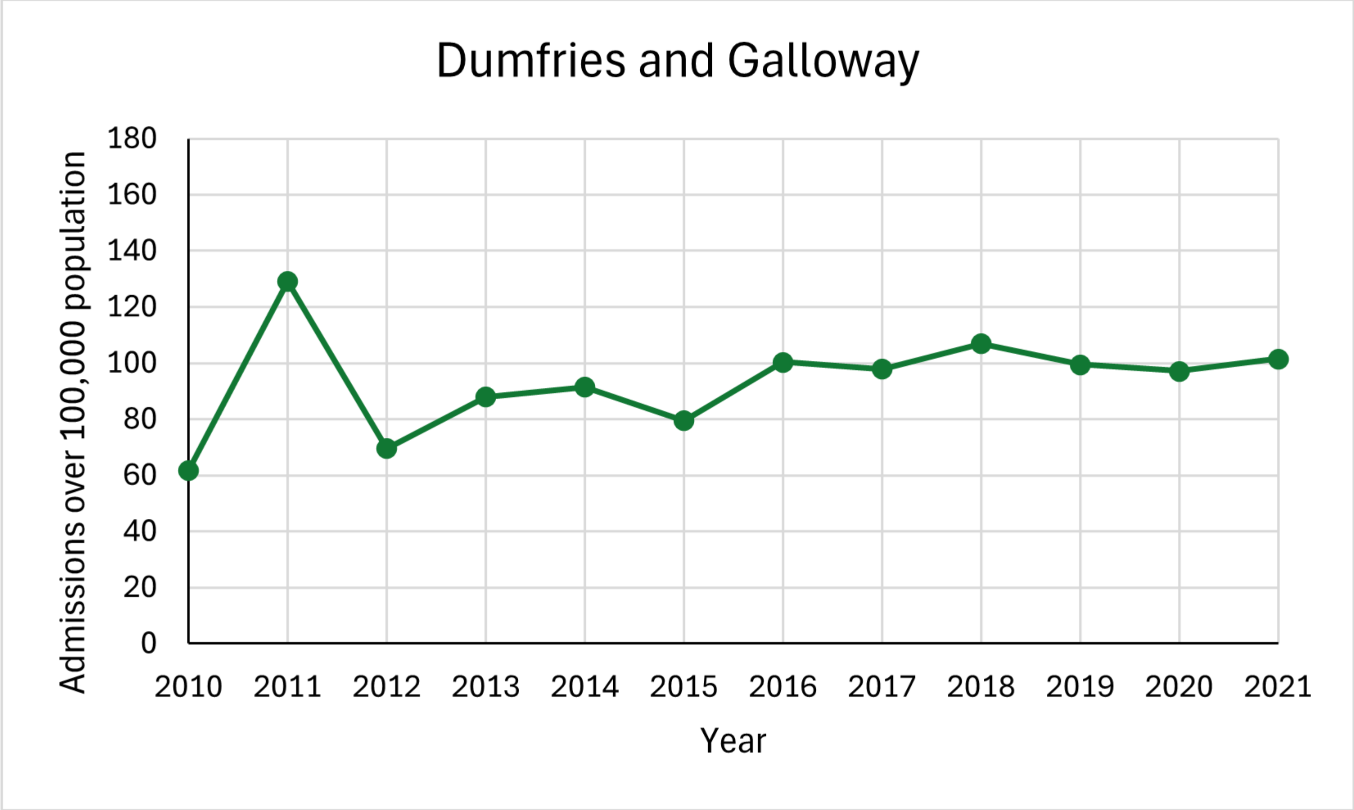

Mean: 93
Median: 97
Standard Deviation: 18
Range: 67

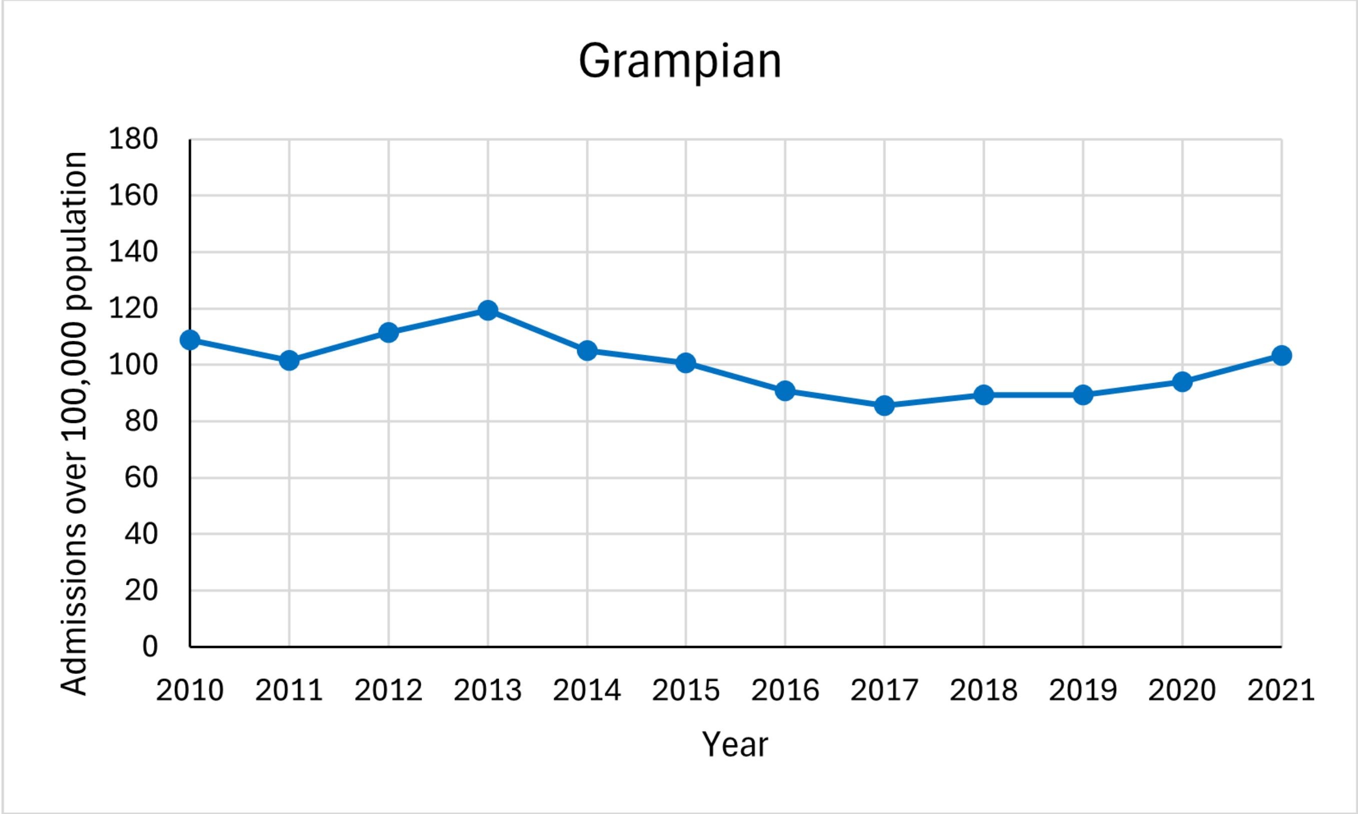

Mean: 100
Median: 101
Standard Deviation: 10
Range: 34

Supplementary file 2: Hospital admissions for paracetamol overdoses in Scotland from 2010 to 2021 by Health Board – Cont'd

This supplementary file show data on hospital admissions for paracetamol overdoses (accidental and intentional) in each Health Board (HB) in Scotland from 2010 to 2021. Values are expressed in ratio of admissions per 100,000 population in the specific HB. Mean, Median, Standard Deviation, Range are also reported for each HB

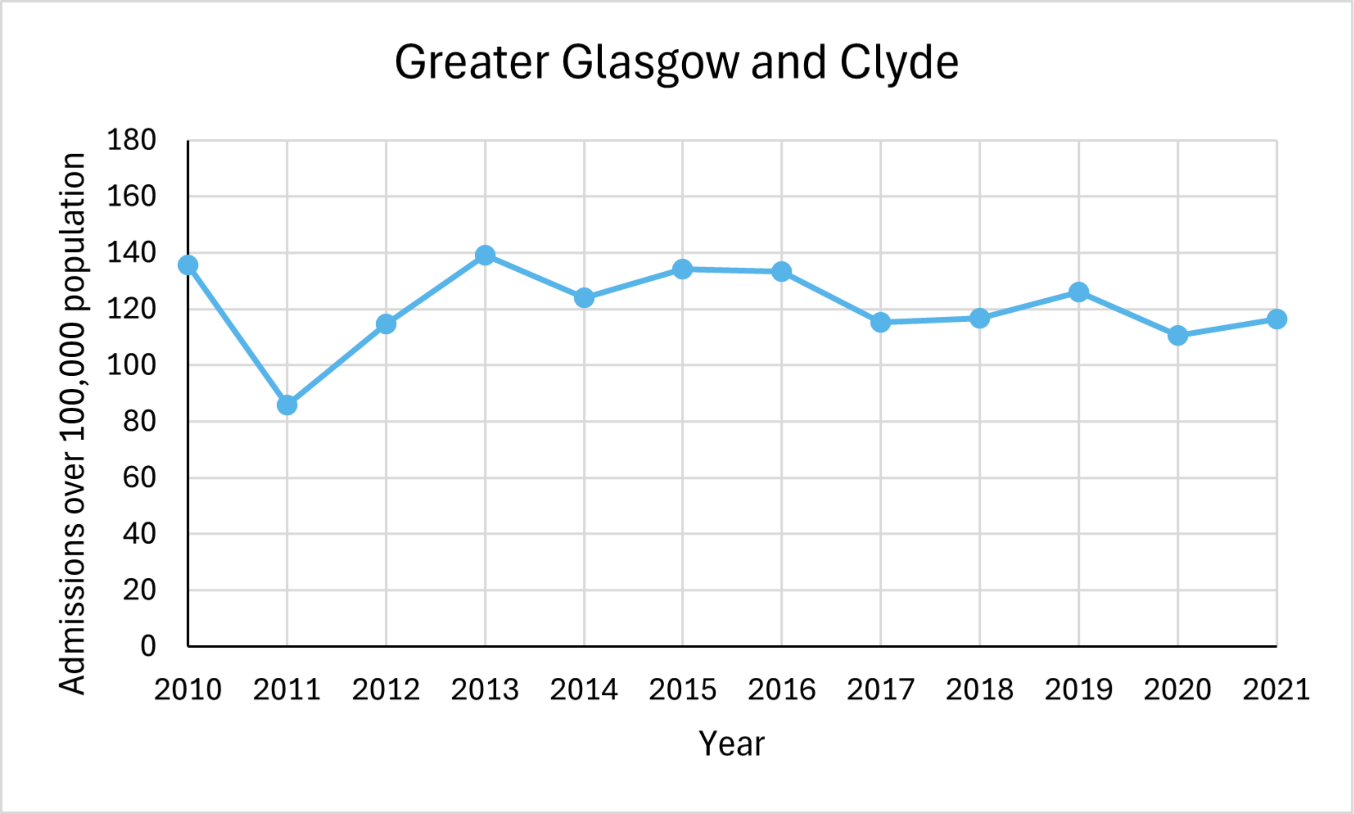

Mean: 121
Median: 120
Standard Deviation: 15
Range: 53

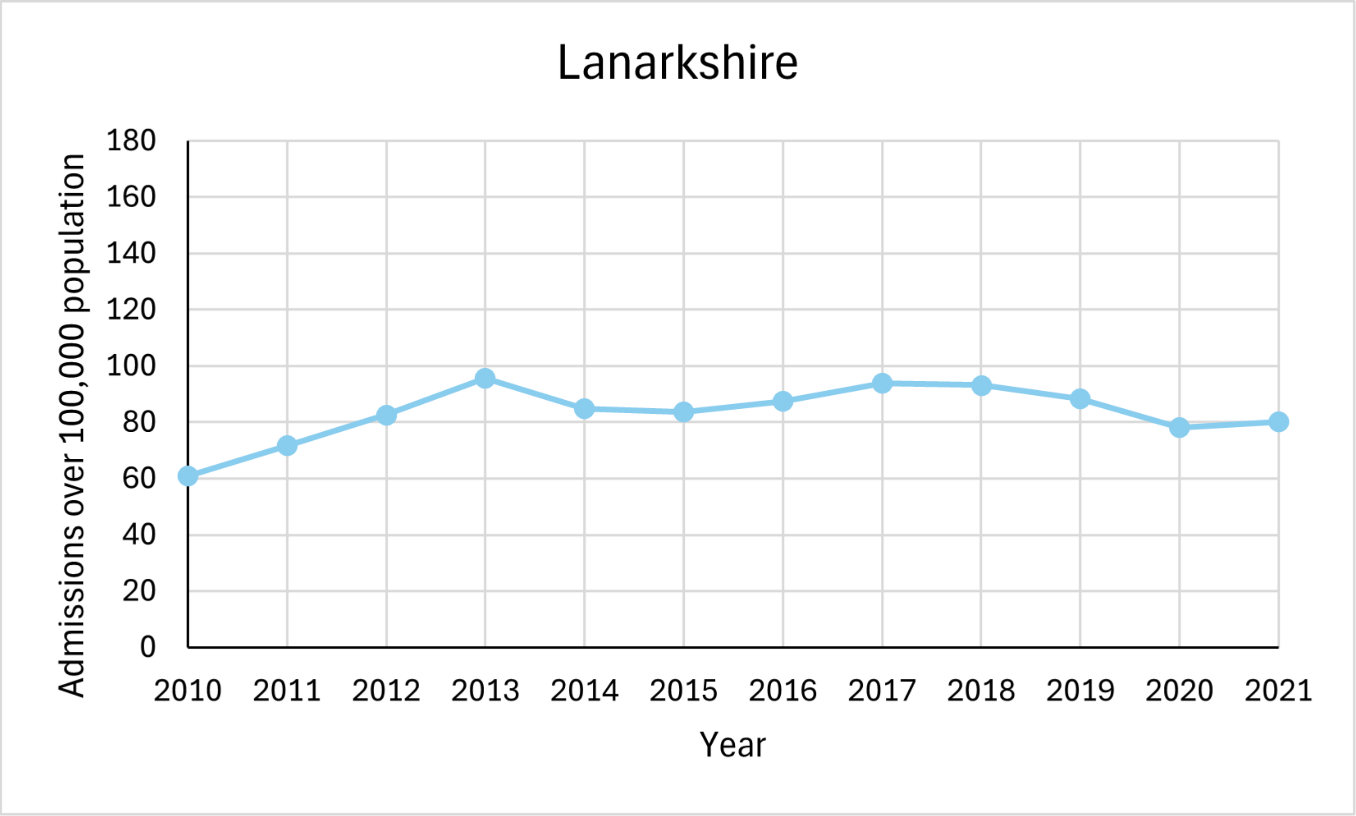

Mean: 83
Median: 84
Standard Deviation: 10
Range: 35

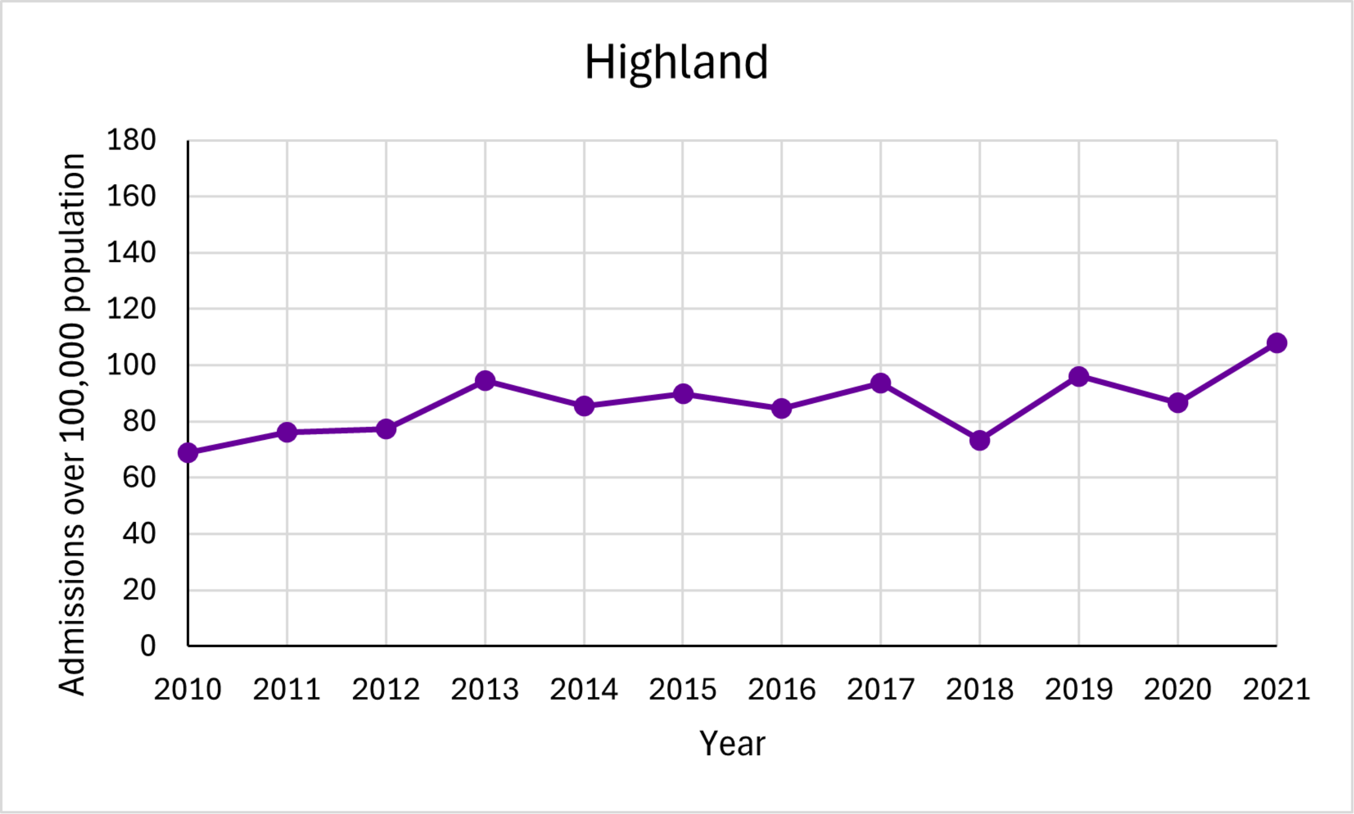

Mean: 86
Median: 86
Standard Deviation: 11
Range: 39

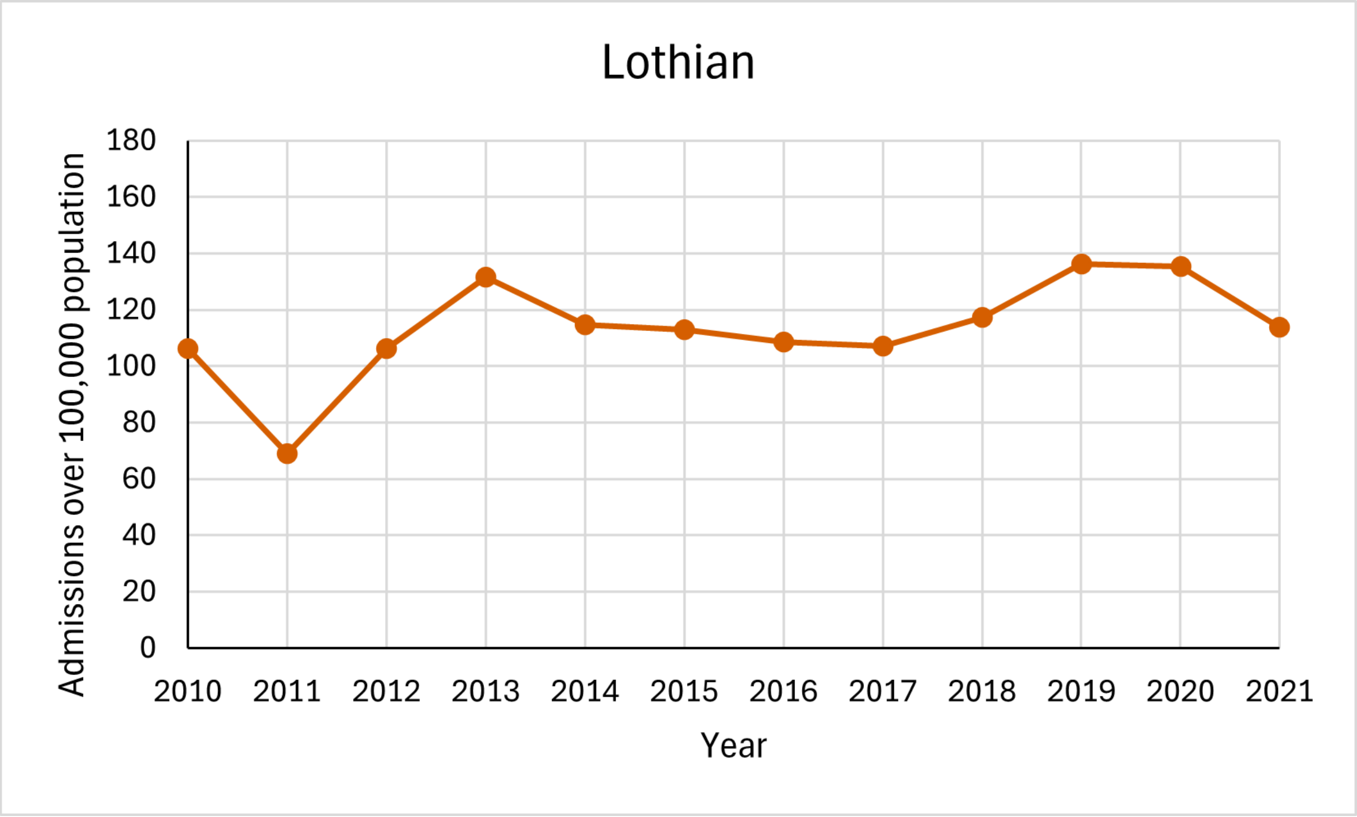

Mean: 113
Median: 113
Standard Deviation: 18
Range: 67

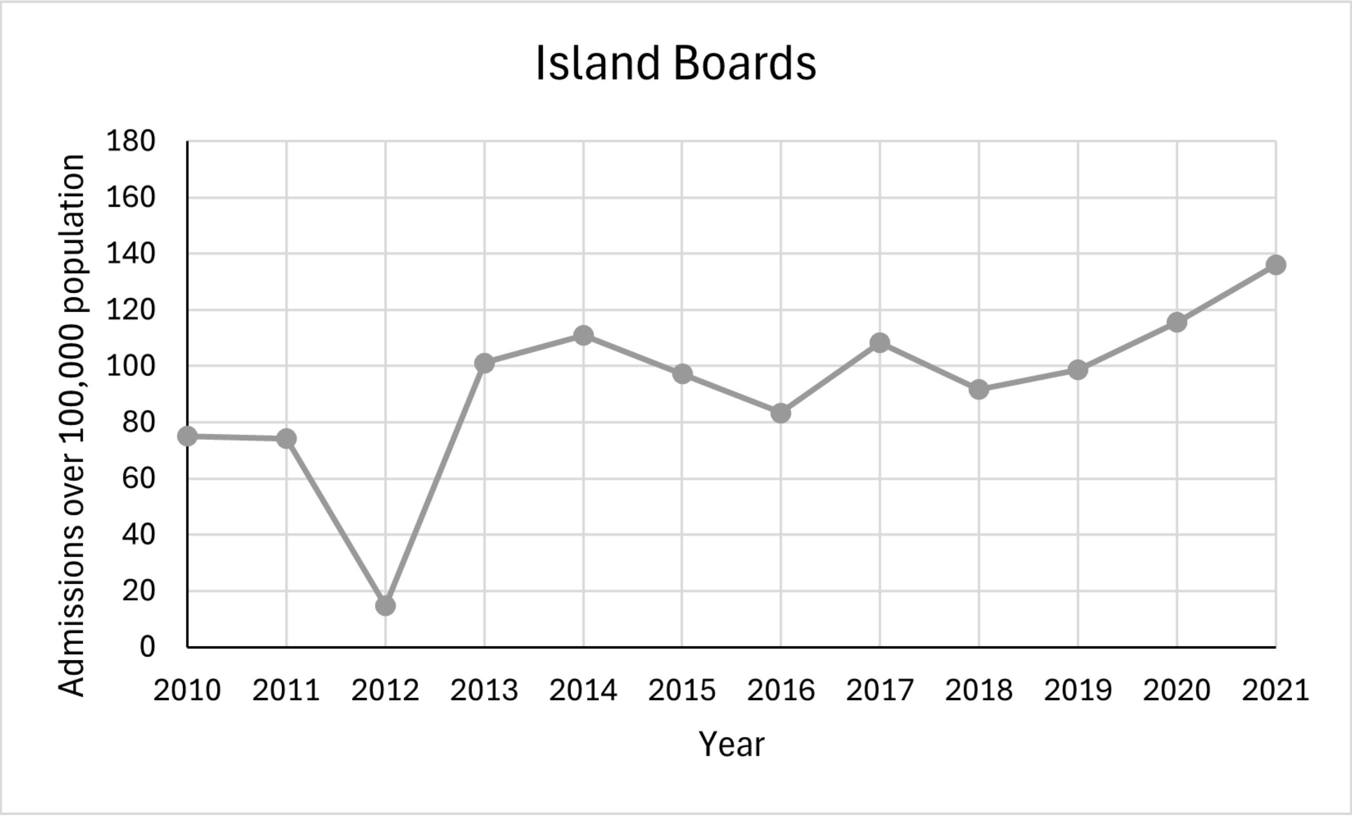

Mean: 92
Median: 98
Standard Deviation: 30
Range: 121

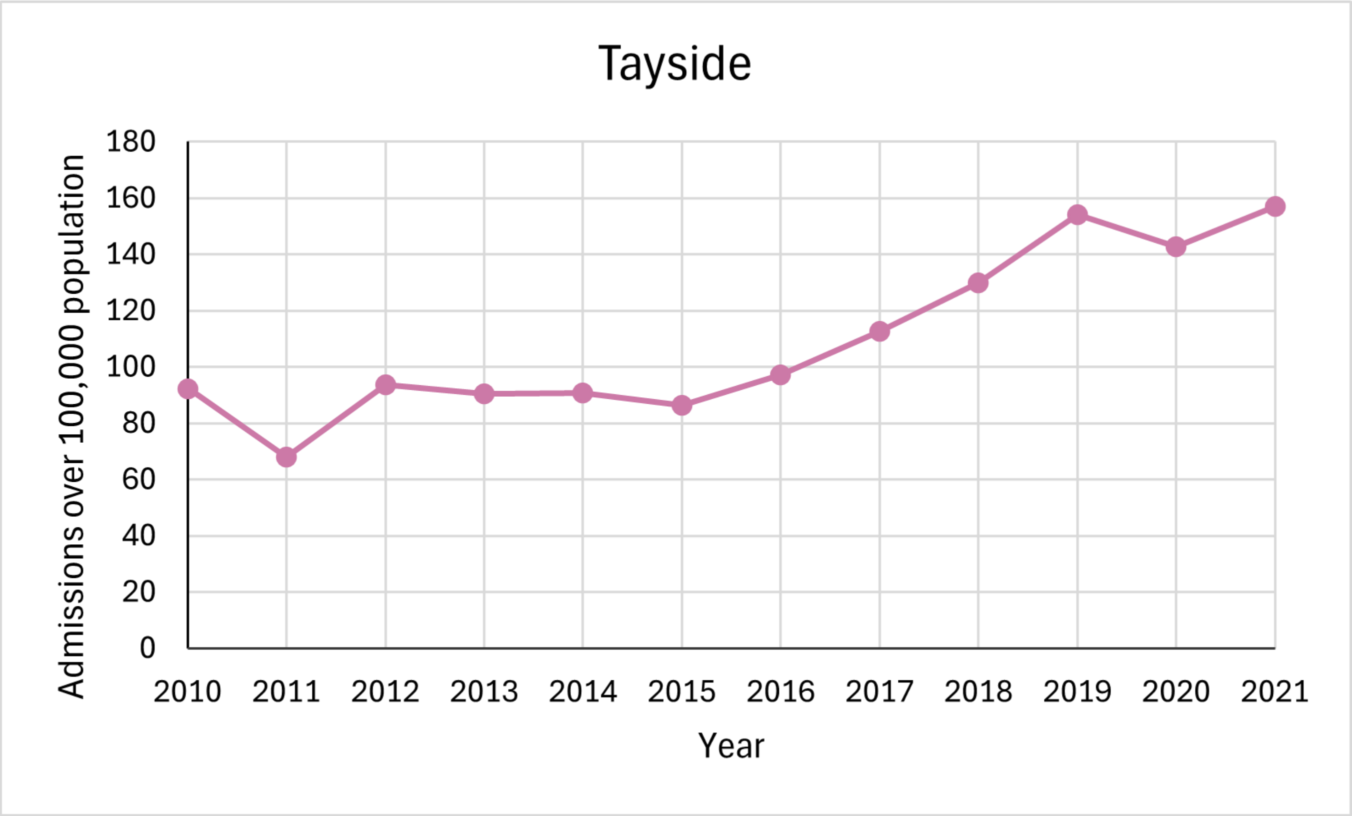

Mean: 110
Median: 95
Standard Deviation: 29
Range: 89
