# Supplementary material for: Still dealing with paracetamol overdoses: epidemiology and quality of data collected in the Scottish health system from 2010 to 2023
Source: J Public Health (Oxf). 2025 Jul 4;47(4):721–7. doi: 10.1093/pubmed/fdaf076 (PMC12669989; doi:10.1093/pubmed/fdaf076)
Supplement: Supplementary_text_fdaf076 [file supplementary_text_fdaf076.docx]

**Supplementary file 1:** Freedom of Information (FOI) responses received from each Health Board are reported with text or tables.

**Supplementary File 2:** Figure with line charts showing paracetamol overdose hospital admissions (per 100,000 population) across Scottish Health Boards from 2010 to 2021. Below each chart, the mean, median, standard deviation and range of annual admissions rates are reported.
